# Is the Use of Artificial Sweeteners Beneficial for Patients with Diabetes Mellitus? The Advantages and Disadvantages of Artificial Sweeteners

**DOI:** 10.3390/nu14214446

**Published:** 2022-10-22

**Authors:** Katsumi Iizuka

**Affiliations:** Department of Clinical Nutrition, Fujita Health University, Toyoake 470-1192, Japan; katsumi.iizuka@fujita-hu.ac.jp; Tel.: +81-472-93-2329

**Keywords:** microbiota, saccharin, sucralose, acesulfame K, aspartame

## Abstract

Artificial sweeteners have been developed as substitutes for sugar. Sucralose, acesulfame K (ACE K), aspartame, and saccharin are artificial sweeteners. Previously, artificial sweeteners were thought to be effective in treating obesity and diabetes. Human meta-analyses have reported that artificial sweeteners have no effect on body weight or glycemic control. However, recent studies have shown that artificial sweeteners affect glucose absorption in the intestinal tract as well as insulin and incretin secretion in humans and animals. Moreover, artificial sweeteners alter the composition of the microbiota and worsen the glycemic control owing to changes in the gut microbiota. The early intake of ACE K was also shown to suppress the taste response to sugar. Furthermore, a large cohort study showed that high artificial sweetener intake was associated with all-cause mortality, cardiovascular risk, coronary artery disease risk, cerebrovascular risk, and cancer risk. The role of artificial sweeteners in the treatment of diabetes and obesity should be reconsidered, and the replacement of sugar with artificial sweeteners in patients will require the long-term tracking of not only intake but also changes in blood glucose and weight as well as future guidance based on gut bacteria data. To utilize the beneficial properties of artificial sweeteners in treatment, further studies are needed.

## 1. Introduction

Artificial sweeteners have been developed as sugar substitutes [1]. Many of these have a much stronger sweetness than simple sugar and sucrose and have few calories [1]. Compared to sucrose, artificial sweeteners are hundreds of times sweeter, so the use of various artificial sweeteners together can reduce the amount of sugar used. Acesulfame K (ACE K), aspartame, and sucralose are widely used and are well-known as artificial sweeteners and are now being studied by many researchers [2]. ACE K and aspartame are used in soft drinks such as soda and protein drinks [2]. The use of artificial sweeteners is thought to prevent dental caries and obesity by reducing the use of sugar, and so-called artificially sweetened beverages are sometimes used in place of sugar-laden soft drinks for obese patients with diabetes mellitus [3]. Therefore, artificial sweeteners are included in many “sweets” and are anticipated to be a “sugar” substitute in patients with diabetes mellitus [3]. However, substituting sugar-sweetened food and beverages with those that have been artificially sweetened may not be as beneficial as once thought [4]. Recently, it was reported that artificial sweeteners can affect glucose tolerance through changes in the microbiota composition [5,6,7,8]. Moreover, artificial sweeteners have side effects in terms of obesity, cardiovascular disease, and mortality [9,10,11,12].

In this review, the kinds of artificial sweeteners and the effect of artificial sweeteners on metabolic effects through sweet taste receptors will be described, and whether artificial sweeteners may be beneficial for diet therapy against diabetes mellitus will be discussed.

## 2. Artificial Sweeteners and Metabolism

### 2.1. Artificial Sweeteners

Currently, several artificial sweeteners are used as food additives [1]. Aspartame, ACE K, sucralose, saccharin, neotame, and advantame are used worldwide as artificial sweeteners. Xylitol, sorbitol, and erythritol belong to another group of sugar alcohols or plant-derived sweeteners. This review will discuss artificial sweeteners.

Aspartame is composed of methanol and two amino acids (aspartate and tryptophan) [1,13,14]. Aspartame is two hundred times sweeter than sucrose [1,13,14]. Aspartame is included in tabletop sweeteners, chewing gums, instant coffee, puddings, and soft drinks. Unlike ACE K and saccharin, aspartame has no bitter aftertaste. Aspartame is labile under heat and breaks down into amino acids. Unlike other artificial sweeteners, aspartame is metabolized into methanol, aspartic acid, and tryptophane [14]. Therefore, aspartame produces 4 kcal of energy per gram when metabolized; however, the quantity of aspartame needed to produce a sweet taste is so small that its caloric contribution is negligible.

Neotame and advantame are aspartame analogs [15,16]. Unlike aspartame, neotame and advantame are non-calorie sweeteners. Neotame (*N*-[*N*-(3,3-dimethylbutyl)-l-α aspartyl]-l-phenylalanine 1-methyl ester) is one of five FDA-approved artificial sweeteners that are 7000–13,000 times sweeter than sugar. Neotame can be metabolized by esterase into de-esterified neotame and methanol and eliminated in the urine and feces within 72 h. Advantame is formally a secondary amine of aspartame and 3-(3-hydroxy-4-methoxyphenyl) propanal (HMPA). A total of 89% of the ingested advantame is excreted in feces, and 6.2% is excreted in urine. Interestingly, advantame is also a flavor enhancer for dairy, fruit, citrus, mint, etc. Advantame is involved in milk products, frozen dairy, nonalcoholic beverages, and chewing gums.

ACE K is present in tabletop sweeteners, carbonated beverages, frozen desserts, candies, chewing gum, dairy products, syrups, and sauces [1,13,14]. ACE K is the potassium salt of 6-methyl-1,2,3-oxathiazine-4(3H)-one 2,2-dioxide. ACE K is a heat-stable sweetener and it is 200 times sweeter than sucrose. It has a bitter aftertaste at high concentrations and is blended with sucralose or aspartame. In fact, its combination with other sweeteners has shown synergistic effects on sweetness. ACE K is not metabolized in vivo and is excreted through the kidney [1,13,14]. Interestingly, it has been reported that the measurement of ACE K is also used for aquatic environmental monitoring [17]. ACE K is involved in dairy products, bakery products, ice cream, tabletop sweeteners, sauces, soups, and processed fishery products.

Saccharin is the oldest artificial sweetener and is 300 times sweeter than sucrose [1,13,14]. Saccharin is also heat-stable. Saccharin is included in soft drinks, fruit drinks, chewing gum, baked goods, and canned fruits. Saccharin is also not metabolized after ingestion and is excreted through the kidneys.

Sucralose (1,6-dichloro-1,6-dideoxy-β-D-fructofuranosyl-4-chloro-4-deoxy-α-D-galactopyranoside) is the most commonly used artificial sweetener [1,13,14]. Sucralose is produced by the chlorination of sucrose (trichlorinated derivative of sucrose). Sucralose is 600 times sweeter than sucrose. Sucralose is included in tabletop sweeteners, baked goods, frozen desserts, fruit juices, chewing gum, and dairy products. Sucralose is water soluble and stable under heat. Sucralose is not metabolized in the body, so it is non-caloric. The majority of sucralose is excreted into feces, and 11–27% is absorbed and excreted in urine.

Table 1 summarizes the characteristics of the artificial sweeteners.

### 2.2. Artificial Sweeteners and Taste Receptors

**Figure 1:**
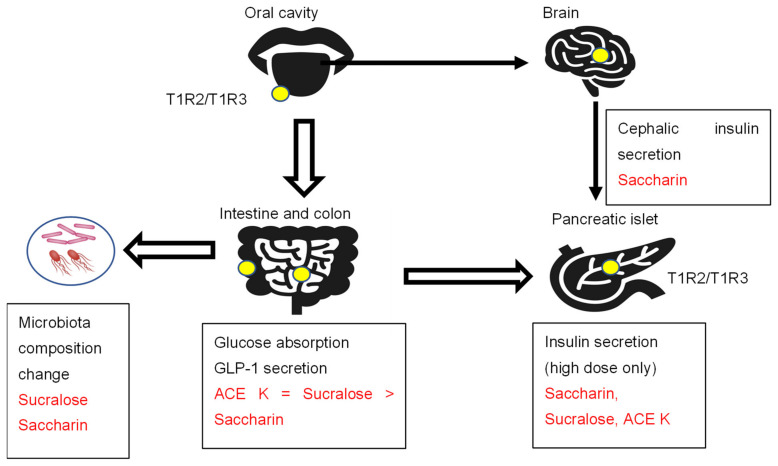
The sweet taste receptors T1R2/T1R3, artificial sweeteners, and metabolism.

Sweet and umami taste receptor signals are transduced by a family of three G protein-coupled receptors: T1R1, T1R2, and T1R3 [18]. The bitter taste receptor is composed of T2R31 or T2R43 [19]. T1R2/T1R3 is expressed in the oral cavity, gastrointestinal tract, pancreas, bladder, adipose tissue, and brain (Figure 1) [18,19].

The sweet taste receptor complex T1R2/T1R3 is expressed in the oral cavity and extraoral tissues, such as the intestine, colon, pancreas, and brain. Artificial sweeteners activate sweet taste receptors and show several effects, such as insulin and incretin secretion and intestinal glucose absorption.

#### 2.2.1. Bitter Aftertaste

Some artificial sweeteners, such as saccharin and ACE K, have a bitter aftertaste, while sucralose and aspartame have no bitter aftertaste. Saccharin and cyclamate are both agonists for the sweet taste receptors TAS1R2 and TAS1R3, although they bind at different sites [19]. Saccharin is also an agonist for the bitter taste receptors TAS2R31 and TAS2R43 [19]. Cyclamate, another artificial sweetener, potently blocks receptors for saccharin’s bitter aftertaste in a dose-responsive manner. Thus, cyclamate acts as a competitive antagonist for these two receptors. These data suggest that combining some artificial sweeteners is beneficial to mask a bitter aftertaste.

#### 2.2.2. Cephalic Phase Insulin Secretion

The initial release of insulin in response to food stimuli acting on receptors in the head and oropharynx is called the cephalic phase of insulin secretion [20]. These physiological insulin responses last for 10 min. A study was conducted on fasted healthy human subjects who washed out their mouths with eight taste solutions (sucrose, saccharin, acetic acid, sodium chloride, quinine hydrochloride, distilled water, starch, and sodium glutamate) for 45 s and then spat them out [21]. The taste stimuli were not swallowed, and they were applied in a randomized order, each on a separate day. Blood collection for the determination of plasma glucose and plasma insulin concentrations was performed 3 min before and 3, 5, 7, and 10 min after taste stimulation. A significant increase in plasma insulin concentration was apparent after stimulation with sucrose and saccharin. Only saccharin stimulated a cephalic-phase insulin response [21]. In many studies, plasma glucose levels are not always checked within 15 min. Since blood glucose and insulin are rarely checked within 15 min after loading, this seems to be an important finding when considering the effects of artificial sweeteners.

#### 2.2.3. Insulin Secretion

Sweet taste receptors are expressed in islet cells [22,23]. The physiological dose of artificial sweeteners (50 μM saccharin) did not affect insulin secretion in rat isolated perfused pancreases [24]. However, a high dose of artificial sweeteners (50 mM saccharin, 50 mM sucralose, or 50 mM ACE K) augmented insulin secretion through taste receptor signaling activation [22]. The artificial sweetener saccharin (50 mM) induced the sustained elevation of [cAMP]i but did not increase [Ca2+]i. In contrast, sucralose (50 mM) and ACE K (50 mM) induced rapid and sustained increases in both [Ca2+]i and [cAMP]i [23]. The potency of insulin secretion in MIN6 cells was ACE K > saccharin = sucralose. Moreover, the genetic ablation of T1R2 suppressed the glucose-stimulated insulin secretion by fructose. These results suggest that sweet taste receptors are functional in pancreatic beta cells. Moreover, these results also suggest that the artificial sweetener-induced metabolic phenotypes may be dependent on the amounts of artificial sweeteners, which are consistent with human data establishing that artificial sweeteners do not affect insulin levels due to the much lower intake compared to sugar.

#### 2.2.4. Intestinal Glucose Absorption and Incretin Secretion

The sweet taste receptors T1R2 and T1R3 are also expressed in the intestine and colon. However, the in vivo effects of glucose metabolism and incretin secretion were inconsistent. Some reported that dietary sugar and artificial sweeteners (2 mM sucralose) increased SGLT1 mRNA and protein expression as well as glucose absorptive capacity in wild-type mice but not in knockout mice lacking T1R3 or alpha-gustducin [25].

GLP-1 and GLP-2 are secreted in a 1:1 ratio by enteroendocrine L cells, most of which are located in the distal ileum and colon [26]. GLP-1 analogs have been developed to treat type 2 diabetes mellitus. In contrast, GLP-2 analogs have been developed to treat gastrointestinal (GI)-related disorders, such as short bowel syndrome (SBS), inflammatory bowel disease (IBD), and chemotherapeutically induced GI mucositis, largely due to the intestinotrophic effects of GLP-2 in the GI tract [26]. In rats, artificial sweeteners (ACE K = sucralose > saccharin) increase glucose absorption in parallel with their ability to increase intracellular calcium concentrations [27]. The intraduodenal infusion of sucralose (0.5 g/kg BW) stimulates GLP-2 secretion in humans. Similarly, sucralose (4%) and, to a lesser extent, saccharin stimulated GLP-2 secretion in rats. In contrast, the intraduodenal infusion of sucralose did not increase intestinal glucose absorption or GLP-1 secretion in healthy subjects [28]. Another study reported that the oral injection of sucralose (1 g/kg BW) and ACE K (1 g/kg BW) did not increase GLP-1 secretion [29].

In recent years, there have been many important reports on the relationship between intestinal microbiota and artificial sweeteners [6]. In rats and mice, aspartame, saccharin, and sucralose were reported to affect the composition of the microbiota.

Some studies have reported that a daily repeated consumption of pure aspartame or sucralose in doses reflective of a typical high consumption (a standardized dose of 14% (0.425 g) of the acceptable daily intake (ADI) for aspartame and 20% (0.136 g) of the ADI for sucralose) for two weeks did not affect the gut microbiota composition or SCFA production in 14 healthy subjects [30].

Recently, some studies have reported that artificial sweeteners worsen glucose clearance in mice and humans [5,7]. In mice, only one week of saccharin, sucralose, and aspartame administration caused glucose intolerance. Saccharin also promoted high-fat diet-induced glucose intolerance. An antibiotic treatment ameliorated the saccharin-induced glucose intolerance in obese mice, which was independent of the mouse strain [5]. Mouse recipients of the saccharin-associated microbiome became glucose intolerant [5]. These results suggest that changes in the gut microbiota induced by saccharin decreased the glucose clearance capacity.

In humans, saccharin (upper limit of the ADI) also promoted glucose intolerance and gut microbiome alterations in four of seven healthy subjects. The microbiome composition of responders and non-responders was different. Thus, the microbiota plays an important role in the development of saccharin-induced glucose intolerance [5]. Moreover, the authors tested whether the ingestion of 0.18 g of saccharin, 0.102 g of sucralose, or 0.24 g of aspartame at the lower dose of the ADI for two weeks caused glucose intolerance in 120 healthy subjects. Saccharin and sucralose-impaired glucose intolerance in healthy subjects. A significant effect on the microbiome composition was observed in the sucralose and saccharin groups. These results suggest that preexposure affected the individual microbiome heterogeneity at baseline, the relationship between the host and the microbiome, and the sucralose-induced glucose intolerance [7].

Perhaps in the future, the evaluation of the role of artificial sweeteners on intestinal bacteria and phenotypes will need to include the distribution of intestinal bacteria before administration, genetic traits (polymorphisms in sugar absorption capacity and taste genes, DNA methylation, etc.), and the duration of artificial sweetener preexposure [31,32].

### 2.3. Body Weight Gain and Diabetes

The effects of artificial sweeteners on glucose metabolism have been discussed. However, there is inconclusive evidence regarding the effects of nonnutritive sweetener (NNS) consumption compared with either sugar, placebo, or nutritive low-calorie sweetener consumption on the clinically relevant benefit or harm to hemoglobin A1c (HbA1c), body weight, and adverse events in people with type 1 or type 2 diabetes [33].

ACE K (0.05%)-containing water caused both body weight gain and fat gain compared to 10% sucrose-sweetened water due to an improved energy efficiency in Sprague Dawley rats (5–6-week-old males) [34]. These effects were independent of drinking water temperature. Energy intake and expenditure were not changed. Lean body mass, plasma glucose, and insulin levels were not changed. Similarly, ACE K (37.5 mg/kg body weight/day) caused body weight gain with changes in microbiota-derived metabolites in male CD-1 mice [35]. These results support the theory that ACE K induces body weight gain. Unfortunately, ACE K (15 mg/kg BW) consumption in 4-week-old rats in the juvenile and adolescent periods did not affect body weight or total calorie intake in male rats. The glucose tolerance test and the gut microbiome were unaffected by ACE K; however, chronic ACE K consumption suppressed sugar taste responsiveness and reduced lingual sweet taste receptor expression. The hippocampal-dependent memory was also impaired [36].

In Sprague–Dawley rats (7-week-old males), the ingestion of 0.05% aspartame significantly increased body weight and fat mass mainly due to an increase in energy efficiency [37]. Energy intake was not changed. These changes are dependent on the amount and independent of the form (solid vs. liquid). Additionally, aspartame ingestion was associated with glucose intolerance and insulin resistance. Sucralose ingestion at 0.016% had a similar impact to that of aspartame, although to a lesser extent [37].

Saccharin was associated with body weight gain. The administration of 0.3% saccharin or 0.4% aspartame over 12 weeks promoted a greater weight gain in adult Wistar rats. This weight gain was unrelated to caloric intake. The long-term intake of saccharin decreases the post-absorptive energy expenditure at rest and is associated with greater weight gain relative to sucrose in Wistar rats [38]. Similarly, saccharin induces weight gain without increasing caloric intake, which is not related to insulin resistance in Wistar rats [39].

Sucralose also enhanced high-fat-diet (HFD)-induced hepatic steatosis [40]. In addition, treatment with sucralose increased reactive oxygen species (ROS) generation and induced endoplasmic reticulum (ER) stress in HepG2 cells. Lipogenic effects are cancelled by pretreatment with taste receptor type 1 membrane 3 (T1R3) inhibitor or T1R3 knockdown in HepG2 cells. Sucralose may activate T1R3 to generate ROS, promote ER stress and lipogenesis, and further accelerate the development of hepatic steatosis. However, as most sucralose is not absorbed in the gut and excreted into the feces, whether sucralose directly affects hepatic lipid metabolism through T1R3 activation remains unclear.

### 2.4. Lipid Metabolism

For aspartame, a meta-analysis of randomized clinical trials showed that the total cholesterol and triglycerides were not affected by aspartame intake compared to the controls or sucrose. However, the serum levels of high-density lipoprotein cholesterol were higher with aspartame compared to the controls (−0.03 mmol/L; 95% CI, −0.06 to −0.01) and lower with aspartame compared to sucrose (0.05 mmol/L; 95% CI, 0.02 to 0.09) [41]. In addition, the treatment of C57BL/6J mice with ACE K for 40 weeks increased not only LDL cholesterol but also HDL cholesterol [42]. A high-dose treatment (final 25, 50, and 100 mM) of AS (aspartame, ACE K, and saccharin) on human high-density lipoprotein (HDL) induced the loss of antioxidant capacity as well as increased atherogenic effects [43]. These results suggest that artificial sweeteners increase plasma HDLc, but AS treatment may impair the beneficial function of HDL [43]. Similarly, the long-term treatment of apoA-I with sweeteners (aspartame, ACE K, saccharin) at physiological concentrations (3 mM, 168 h) resulted in the loss of antioxidant and phospholipid binding activities and the modification of the secondary structure [44] (application of the antioxidant and phospholipid binding activities with modification of the secondary structure). AS-treated apoA-I also underwent proteolysis, producing a 26 kDa fragment. These findings suggest that artificial sweeteners impair the antiatherogenic effect of HDLc by modifying HDL, and in particular apoA-1 [44].

Interestingly, clofibrate inhibits sweet taste receptor activity by binding to T1R. Furthermore, clofibrate was found to inhibit the perception of sweetness in four artificial sweeteners: sucrose, sucralose, sodium cyclamate, and ACE K [45]. These findings suggest that several lipid-lowering drugs may influence sweet taste preference.

Fibroblast growth factor (FGF21) is a known hormone that regulates glucose and lipid metabolism [45]; FGF21 is a hepatokine secreted by the liver [46]. The administration of FGF21 or its analogs to obese nonhuman primates has been shown to decrease food intake, reduce overweight, and improve plasma lipid profiles while increasing circulating adiponectin [47,48]. In addition, FGF21 acts on the paraventricular nucleus of the hypothalamus to suppress carbohydrate intake and carbohydrate preference, which correlates with the decreased dopaminergic neurotransmission within the nucleus accumbens [49,50,51]. Consistently, the acute administration or overexpression of FGF21 suppresses the intake of both sugar and non-caloric sweeteners. On the other hand, glucose and fructose can induce Fgf21 mRNA levels, whereas saccharin could not induce FGF21 mRNA [49]. I previously reported that FGF21 expression is regulated by glucose-activated transcription factors, carbohydrate response element binding proteins, and carbohydrate response element binding proteins (ChREBPs) [52]. ChREBPs regulate genes in the lipogenesis, glycogenesis, glycolysis, and pentose phosphate cycle pathways regulated by ChREBP [53]. Glucose activates ChREBP by increasing glucose 6-phosphate and xylulose 5-phosphate, but artificial sweeteners failed to activate ChREBP because they did not alter the levels of the glucose-derived metabolites. Artificial sweeteners do not activate ChREBP [53]; FGF21 mediates the endocrine regulation of simple carbohydrate intake and sweet taste preference by the liver, so the intake of artificial sweeteners does not suppress sweet intake [49]. These results suggest that the FGF21-mediated negative feedback pathway to abstain from sugar intake, as seen with other monosaccharides, does not apply to artificial sweeteners.

### 2.5. Cardiovascular Disease, Cancer Incidence, and Mortality

Previously, the relationship between artificial sweeteners and cardiovascular risk was unclear. In a women’s health initiative study, a higher intake of artificially sweetened beverages was associated with an increased risk of stroke, coronary heart disease, and all-cause mortality; however, a higher intake of artificially sweetened beverages was not associated with hemorrhagic stroke [10].

The detailed mechanism as to why cerebral infarction, but not cerebral hemorrhage, increases is unclear. Experiments using cholesterol-loaded ApoE knockout mice have been reported to promote atherosclerosis. In these mice, cholesterol loading increased the blood total cholesterol and triglyceride levels, increased low-density lipoprotein (LDL) cholesterol, and decreased high-density lipoprotein (HDL) cholesterol; increased hepatic SREBP1 expression by ACE K was accompanied by increased Fasn and Acc1 and decreased Acox, suggesting that ACE K consumption with dietary modifications may result in the worsening of lipid abnormalities and the development of atherosclerosis [11].

An important report was very recently published. This study was conducted on 103,388 participants in the web-based NutriNet cohort [9]. This study examined aspartame, ACE K, and sucralose, but not saccharin. The total artificial sweetener intake was associated with an increased cardiovascular risk (hazard ratio 1.09, 95% confidence interval 1.01–1.18, *p*=0.03). Consistently with a previous study, the total artificial sweetener intake was associated with cerebrovascular disease (HR 1.18, 95% CI 1.06–1.31, *p* = 0.002). ACE K and sucralose were associated with coronary heart disease risk (ACE K: 1.40, 1.06–1.84, *p* = 0.02; sucralose: 1.31, 1.00–1.71, *p* = 0.05); however, aspartame was not associated with coronary heart disease risk (0.91, 0.78–1.06, *p* = 0.49). Aspartate intake was associated with an increased risk of cerebrovascular disease (aspartame: 1.17, 1.03–1.33, *p* = 0.02), but ACE K and sucralose were not associated with an increased risk of cerebrovascular disease (ACE K: 1.01, 0.79–1.29, *p* = 0.93; sucralose: 0.99, 0.76–1.29, *p* = 0.93). These results suggest no benefit from substituting artificial sweeteners for added sugar on cardiovascular disease (CVD) outcome [9]. It is very interesting and significant that different artificial sweeteners have different risks of heart disease and cerebrovascular disease.

There has long been interest in the relationship between cancer and its known causes, including aspartame and lymphoma in animal studies, cyclamate and bladder cancer, and ACE K and thyroid tumors [13]. The relationship with cancer has also been investigated: 25 observational studies (3,739,775 subjects) found no association between the intake of artificial sweeteners and cancer mortality or incidence, but only European data showed an association with cancer incidence (1.07, 1.02–1.12, *p* = 0.058), as opposed to the United States or Oceania. There was no association with cancer mortality in any of the regions. However, there was an association with total mortality (1.13, 1.03–1.25, *p* < 0.001) [11]. The differences in carcinogenesis rates by region reflect the fact that the effects of artificial sweeteners vary more with individual patients, including the gut bacteria, which are more strongly influenced by lifestyle than the effects of ACE K alone.

In the NutriNet-Sante population-based cohort study, artificial sweeteners (especially aspartame and ACE K) were also associated with increased cancer risk. Higher consumers of total artificial sweeteners had a higher risk of overall cancer (hazard ratio (HR) = 1.13 [95% CI 1.03–1.25], *p* = 0.002). Aspartame (HR = 1.15 [95% CI 1.03–1.28], *p* = 0.002) and ACE K (HR = 1.13 [95% CI 1.01–1.26], *p* = 0.007) were associated with an increased cancer risk. Higher risks were also observed for breast cancer (n = 979 cases, HR = 1.22 [95% CI 1.01–1.48], *p* = 0.036, for aspartame) and obesity-related cancers (n = 2023 cases, HR = 1.13 [95% CI 1.00–1.28], *p* = 0.036, for total artificial sweeteners, and HR = 1.15 [95% CI 1.01–1.32], *p* = 0.026, for aspartame) [12]. This finding might be due to the relationship between obesity and artificial sweeteners.

**Figure 2:**
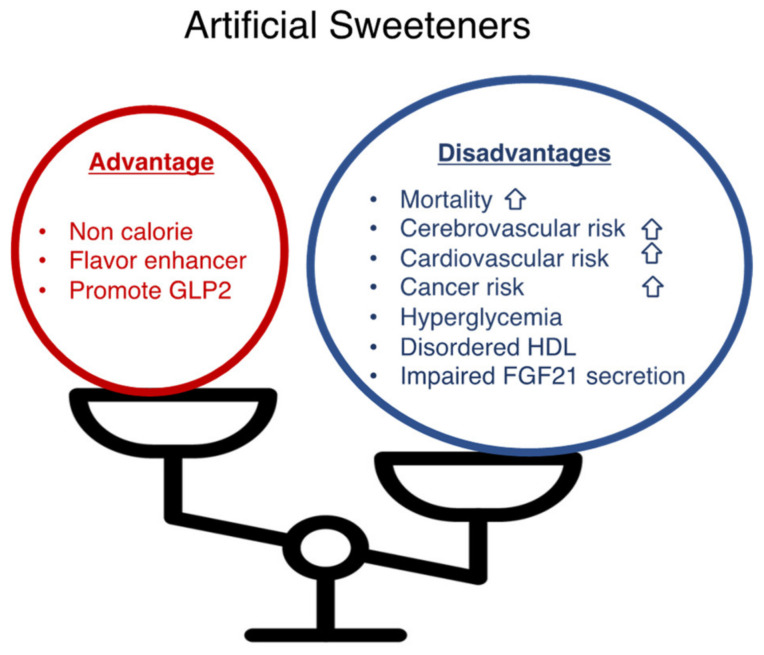
Artificial sweeteners have advantages and disadvantages. Artificial sweeteners (sucralose, saccharin, aspartame, and acesulfam K) present advantages (non-caloric; a flavor enhancer; a GLP-2 stimulant) and disadvantages (increased mortality, cerebrovascular risk, cardiovascular risk, hyperglycemia, increased plasma HDL levels with impaired antioxidant functions, impaired FGF21 secretion).

Finally, I showed the summary of this review (Figure 2).

## 3. Conclusions and Perspective

**Figure 3:**
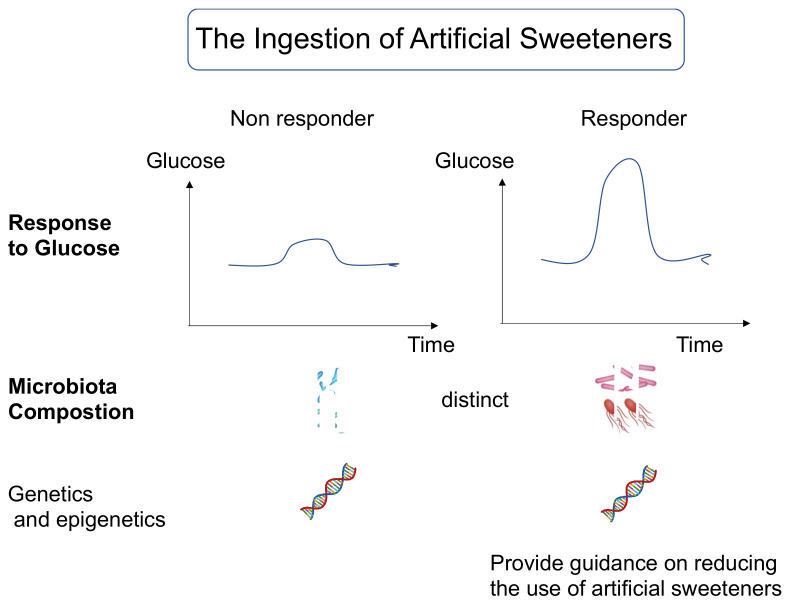
Questioning the intake of artificial sweeteners may be an important issue in nutritional guidance in the near future.

Notably, the effects of artificial sweeteners depend on individual differences, including the gut bacteria, and may increase blood glucose levels, promote atherosclerosis, and increase cardiovascular risk and total mortality. Therefore, the replacement of sugar with artificial sweeteners in patients should be monitored over time for changes in blood glucose and body weight as well as intake, and future guidance should be based on gut bacteria data (Figure 3). It should be noted, however, that the use of artificial sweeteners from an early age may lead to an insensitivity to sweetness, which may increase cardiovascular risk and total mortality.

Understanding individual information, including gut bacteria, genetic traits, and epigenetics, will lead to future risk assessments, such as elevated blood sugar from artificial sweeteners.

It is also interesting to note that the effect of artificial sweeteners on the intestinal tract is to promote GLP-2 secretion without increasing blood flow. GLP-2 is thought to contribute to homeostatic signals such as the promotion of nutrient absorption and the maintenance and repair of the intestinal mucosa; patients with digestive disorders who have residual GLP-2-secreting cells may benefit from short-term artificial sweetener administration. As teduglutide, a GLP-2 analog, is beneficial for parenteral support (PS) reduction in patients with short bowel syndrome, especially Crohn’s disease [54], artificial sweeteners might be effective for the treatment of digestive diseases.

Future research on artificial sweeteners should take into account individual differences, including the intestinal microbiota, the type of artificial sweetener itself, and the duration of previous artificial sweetener administration. In any case, as the effects of artificial sweeteners on intestinal oxygen consumption, the gut microbiota, and incretin secretion become clearer, unexpected uses may be found, such as maintaining and enhancing the intestinal function.

## Figures and Tables

**Table 1:** Characteristics of artificial sweeteners.

	Sweetness Relative to Sucrose by Weight	ADI (mg/kg BW/Day)	Calories	Metabolism	Heat	Bitter Aftertaste
Acesulfame potassium (ACE K)	200	15	0	Excreted through the kidney	Stable	Yes
Aspartame	180–200	50	4	100% absorbed; Metabolized into Methanol + Aspartate +Phenylalanine	Labile	No
Neotame	7000–13,000	18	0	Metabolized into de-esterified neotame and methanol	Stable	No
Advantame	20,000	32.8	0	Excreted in feces	Stable	
Saccharin	300	5	0	85% absorbed; Excreted through kidneys o-sulfamoylbenzoic acid	Stable	Yes
Sucralose	600	5	0	15% absorbed; Excreted into feces	Stable	No

## Data Availability

Not applicable.

## References

[1] Chattopadhyay S., Raychaudhuri U., Chakraborty R. (2014). Artificial sweeteners—A review. J. Food Sci. Technol..

[2] Brown R.J., de Banate M.A., Rother K.I. (2010). Artificial sweeteners: A systematic review of metabolic effects in youth. Int. J. Pediatr. Obes..

[3] Evert A.B., Dennison M., Gardner C.D., Garvey W.T., Lau K.H.K., MacLeod J., Mitri J., Pereira R.F., Rawlings K., Robinson S. (2019). Nutrition Therapy for Adults with Diabetes or Prediabetes: A Consensus Report. Diabetes Care.

[4] Nichol A.D., Holle M.J., An R. (2018). Glycemic impact of nonnutritive sweeteners: A systematic review and meta-analysis of randomized controlled trials. Eur. J. Clin. Nutr..

[5] Suez J., Korem T., Zeevi D., Zilberman-Schapira G., Thaiss C.A., Maza O., Israeli D., Zmora N., Gilad S., Weinberger A. (2014). Artificial sweeteners induce glucose intolerance by altering the gut microbiota. Nature.

[6] Ruiz-Ojeda F.J., Plaza-Díaz J., Sáez-Lara M.J., Gil A. (2019). Effects of Sweeteners on the Gut Microbiota: A Review of Experimental Studies and Clinical Trials. Adv. Nutr..

[7] Suez J., Cohen Y., Valdés-Mas R., Mor U., Dori-Bachash M., Federici S., Zmora N., Leshem A., Heinemann M., Linevsky R. (2022). Personalized microbiome-driven effects of nonnutritive sweeteners on human glucose tolerance. Cell.

[8] Page K.A. (2022). A gut reaction: Microbiome-driven glycemic effects of nonnutritive sweeteners. Cell.

[9] Debras C., Chazelas E., Sellem L., Porcher R., Druesne-Pecollo N., Esseddik Y., de Edelenyi F.S., Agaësse C., De Sa A., Lutchia R. (2022). Artificial sweeteners and risk of cardiovascular diseases: Results from the prospective NutriNet-Santé cohort. BMJ.

[10] Mossavar-Rahmani Y., Kamensky V., Manson J.E., Silver B., Rapp S.R., Haring B., Beresford S.A.A., Snetselaar L., Wassertheil-Smoller S. (2019). Artificially Sweetened Beverages and Stroke, Coronary Heart Disease, and All-Cause Mortality in the Women’s Health Initiative. Stroke.

[11] Yan S., Yan F., Liu L., Li B., Liu S., Cui W. (2022). Can Artificial Sweeteners Increase the Risk of Cancer Incidence and Mortality: Evidence from Prospective Studies. Nutrients.

[12] Debras C., Chazelas E., Srour B., Druesne-Pecollo N., Esseddik Y., Szabo de Edelenyi F., Agaësse C., De Sa A., Lutchia R., Gigandet S. (2022). Artificial sweeteners and cancer risk:Results from the NutriNet-Sante population-based cohort study. PLoS Med.

[13] Whitehouse C.R., Boullata J., McCauley L.A. (2008). The potential toxicity of artificial sweeteners. AAOHN J..

[14] Magnuson B.A., Carakostas M.C., Moore N.H., Poulos S.P., Renwick A.G. (2016). Biological fate of low-calorie sweeteners. Nutr. Rev..

[15] Satyavathi K., Raju P.B., Bupesh K., Kiran T.N.R. (2010). Neotame: High intensity low caloric sweetener. Asian J. Chem..

[16] Wilk K., Korytek W., Pelczyńska M., Moszak M., Bogdański P. (2022). The Effect of Artificial Sweeteners Use on Sweet Taste Perception and Weight Loss Efficacy: A Review. Nutrients.

[17] Buerge I.J., Buser H.R., Kahle M., Müller M.D., Poiger T. (2009). Ubiquitous occurrence of the artificial sweetener acesulfame in the aquatic environment: An ideal chemical marker of domestic wastewater in groundwater. Environ. Sci. Technol..

[18] Laffitte A., Neiers F., Briand L. (2014). Functional roles of the sweet taste receptor in oral and extraoral tissues. Curr. Opin. Clin. Nutr. Metab. Care.

[19] Munger S.D. (2017). A Bitter Tale of Sweet Synergy. Cell Chem. Biol..

[20] Wiedemann S.J., Rachid L., Illigens B., Böni-Schnetzler M., Donath M.Y. (2020). Evidence for cephalic phase insulin release in humans: A systematic review and meta-analysis. Appetite.

[21] Just T., Pau H.W., Engel U., Hummel T. (2008). Cephalic phase insulin release in healthy humans after taste stimulation?. Appetite.

[22] Nakagawa Y., Nagasawa M., Mogami H., Lohse M., Ninomiya Y., Kojima I. (2013). Multimodal function of the sweet taste receptor expressed in pancreatic β-cells: Generation of diverse patterns of intracellular signals by sweet agonists. Endocr. J..

[23] Nakagawa Y., Nagasawa M., Yamada S., Hara A., Mogami H., Nikolaev V.O., Lohse M.J., Shigemura N., Ninomiya Y., Kojima I. (2009). Sweet taste receptor expressed in pancreatic beta-cells activates the calcium and cyclic AMP signaling systems and stimulates insulin secretion. PLoS ONE.

[24] Usami M., Seino Y., Takai J., Nakahara H., Seino S., Ikeda M., Imura H. (1980). Effect of cyclamate sodium, saccharin sodium and stevioside on arginine-induced insulin and glucagon secretion in the isolated perfused rat pancreas. Horm. Metab. Res..

[25] Margolskee R.F., Dyer J., Kokrashvili Z., Salmon K.S., Ilegems E., Daly K., Maillet E.L., Ninomiya Y., Mosinger B., Shirazi-Beechey S.P. (2007). T1R3 and gustducin in gut sense sugars to regulate expression of Na+-glucose cotransporter 1. Proc. Natl. Acad. Sci. USA.

[26] Janssen P., Rotondo A., Mulé F., Tack J. (2013). Review article: A comparison of glucagon-like peptides 1 and 2. Aliment. Pharm. Ther..

[27] Mace O.J., Affleck J., Patel N., Kellett G.L. (2007). Sweet taste receptors in rat small intestine stimulate glucose absorption through apical GLUT2. J. Physiol..

[28] Ma J., Chang J., Checklin H.L., Young R.L., Jones K.L., Horowitz M., Rayner C.K. (2010). Effect of the artificial sweetener, sucralose, on small intestinal glucose absorption in healthy human subjects. Br. J. Nutr..

[29] Fujita Y., Wideman R.D., Speck M., Asadi A., King D.S., Webber T.D., Haneda M., Kieffer T.J. (2009). Incretin release from gut is acutely enhanced by sugar but not by sweeteners in vivo. Am. J. Physiol. Endocrinol. Metab..

[30] Ahmad S.Y., Friel J., Mackay D. (2020). The Effects of Non-Nutritive Artificial Sweeteners, Aspartame and Sucralose, on the Gut Microbiome in Healthy Adults: Secondary Outcomes of a Randomized Double-Blinded Crossover Clinical Trial. Nutrients.

[31] Iizuka K., Yabe D. (2020). The Role of Metagenomics in Precision Nutrition. Nutrients.

[32] Simopoulos A.P. (2010). Nutrigenetics/Nutrigenomics. Annu. Rev. Public Health.

[33] Lohner S., Kuellenberg de Gaudry D., Toews I., Ferenci T., Meerpohl J.J. (2020). Non-nutritive sweeteners for diabetes mellitus. Cochrane Database Syst. Rev..

[34] Ragi M., El-Helou N., El-Mallah C., Eid A., Obeid O. (2021). Effect of temperature and/or sweetness of beverages on body composition in rats. Br. J. Nutr..

[35] Bian X., Chi L., Gao B., Tu P., Ru H., Lu K. (2017). The artificial sweetener acesulfame potassium affects the gut microbiome and body weight gain in CD-1 mice. PLoS ONE.

[36] Tsan L., Chometton S., Hayes A.M., Klug M.E., Zuo Y., Sun S., Bridi L., Lan R., Fodor A.A., Noble E.E. (2022). Early life low-calorie sweetener consumption disrupts glucose regulation, sugar-motivated behavior, and memory function in rats. JCI Insight.

[37] Ragi M., El-Haber R., El-Masri F., Obeid O. (2022). The effect of aspartame and sucralose intake on body weight measures and blood metabolites: Role of their form (solid and/or liquid) of ingestion. Br. J. Nutr..

[38] Evans M., Guthrie N., Pezzullo J., Sanli T., Fielding R.A., Bellamine A. (2017). Efficacy of a novel formulation of L-Carnitine, creatine, and leucine on lean body mass and functional muscle strength in healthy older adults: A randomized, double-blind placebo-controlled study. Nutr. Metab..

[39] Foletto K.C., Melo Batista B.A., Neves A.M., de Matos Feijó F., Ballard C.R., Marques Ribeiro M.F., Bertoluci M.C. (2016). Sweet taste of saccharin induces weight gain without increasing caloric intake, not related to insulin-resistance in Wistar rats. Appetite.

[40] Wu H.T., Lin C.H., Pai H.L., Chen Y.C., Cheng K.P., Kuo H.Y., Li C.H., Ou H.Y. (2022). Sucralose, a Non-nutritive Artificial Sweetener Exacerbates High Fat Diet-Induced Hepatic Steatosis Through Taste Receptor Type 1 Member 3. Front. Nutr..

[41] Santos N.C., de Araujo L.M., De Luca Canto G., Guerra E.N.S., Coelho M.S., Borin M.F. (2018). Metabolic effects of aspartame in adulthood: A systematic review and meta-analysis of randomized clinical trials. Crit. Rev. Food Sci. Nutr..

[42] Cong W.N., Wang R., Cai H., Daimon C.M., Scheibye-Knudsen M., Bohr V.A., Turkin R., Wood W.H., Becker K.G., Moaddel R. (2013). Long-term artificial sweetener acesulfame potassium treatment alters neurometabolic functions in C57BL/6J mice. PLoS ONE.

[43] Kim J.Y., Park K.H., Kim J., Choi I., Cho K.H. (2015). Modified high-density lipoproteins by artificial sweetener, aspartame, and saccharin, showed loss of anti-atherosclerotic activity and toxicity in zebrafish. Cardiovasc. Toxicol..

[44] Jang W., Jeoung N.H., Cho K.H. (2011). Modified apolipoprotein (apo) A-I by artificial sweetener causes severe premature cellular senescence and atherosclerosis with impairment of functional and structural properties of apoA-I in lipid-free and lipid-bound state. Mol. Cells.

[45] Kochem M., Breslin P.A. (2017). Lipid-Lowering Pharmaceutical Clofibrate Inhibits Human Sweet Taste. Chem. Senses.

[46] Geng L., Lam K.S.L., Xu A. (2020). The therapeutic potential of FGF21 in metabolic diseases: From bench to clinic. Nat. Rev. Endocrinol..

[47] Thompson W.C., Zhou Y., Talukdar S., Musante C.J. (2016). PF-05231023, a long-acting FGF21 analogue, decreases body weight by reduction of food intake in non-human primates. J. Pharmacokinet. Pharmacodyn..

[48] Talukdar S., Zhou Y., Li D., Rossulek M., Dong J., Somayaji V., Weng Y., Clark R., Lanba A., Owen B.M. (2016). A Long-Acting FGF21 Molecule, PF-05231023, Decreases Body Weight and Improves Lipid Profile in Non-human Primates and Type 2 Diabetic Subjects. Cell Metab..

[49] Von Holstein-Rathlou S., BonDurant L.D., Peltekian L., Naber M.C., Yin T.C., Claflin K.E., Urizar A.I., Madsen A.N., Ratner C., Holst B. (2016). FGF21 Mediates Endocrine Control of Simple Sugar Intake and Sweet Taste Preference by the Liver. Cell Metab..

[50] Søberg S., Sandholt C.H., Jespersen N.Z., Toft U., Madsen A.L., von Holstein-Rathlou S., Grevengoed T.J., Christensen K.B., Bredie W.L., Potthoff M.J. (2017). FGF21 Is a Sugar-Induced Hormone Associated with Sweet Intake and Preference in Humans. Cell Metab..

[51] Talukdar S., Owen B.M., Song P., Hernandez G., Zhang Y., Zhou Y., Scott W.T., Paratala B., Turner T., Smith A. (2016). FGF21 Regulates Sweet and Alcohol Preference. Cell Metab..

[52] Iizuka K., Takeda J., Horikawa Y. (2009). Glucose induces FGF21 mRNA expression through ChREBP activation in rat hepatocytes. FEBS Lett..

[53] Iizuka K. (2021). The Roles of Carbohydrate Response Element Binding Protein in the Relationship between Carbohydrate Intake and Diseases. Int. J. Mol. Sci..

[54] Bioletto F., D’Eusebio C., Merlo F.D., Aimasso U., Ossola M., Pellegrini M., Ponzo V., Chiarotto A., De Francesco A., Ghigo E. (2022). Efficacy of Teduglutide for Parenteral Support Reduction in Patients with Short Bowel Syndrome: A Systematic Review and Meta-Analysis. Nutrients.

